# Reducing Unplanned Extubations Across a Children’s Hospital Using Quality Improvement Methods

**DOI:** 10.1097/pq9.0000000000000114

**Published:** 2018-12-11

**Authors:** Sarah B. Kandil, Beth L. Emerson, Michael Hooper, Rebecca Ciaburri, Christie J. Bruno, Nancy Cummins, Virginia DeFilippo, Beth Blazevich, Adrienne Loth, Matthew Grossman

**Affiliations:** From the * Department of Pediatrics, Yale School of Medicine, New Haven, Conn.; † Yale New Haven Children’s Hospital, New Haven, Conn.

## Abstract

**Introduction:**

Children who require an endotracheal (ET) tube for care during critical illness are at risk of unplanned extubations (UE), or the unintended dislodgement or removal of an ET tube that can lead to significant patient harm. A proposed national benchmark is 1 UE per 100 ventilator days. We aimed to reduce the rate of UEs in our intensive care units (ICUs) from 1.20 per 100 ventilator days to below the national benchmark within 2 years.

**Methods:**

We identified several key drivers including ET securement standardization, safety culture, and strategies for high-risk situations. We employed quality improvement methodologies including apparent cause analysis and plan-do-study-act cycles to improve our processes and outcomes.

**Results:**

Over 2 years, we reduced the rate of UEs hospital-wide by 75% from 1.2 to 0.3 per 100 ventilator days. We eliminated UEs in the pediatric ICU during the study period, while the UE rate in the neonatal ICU also decreased from 1.2 to 0.3 per 100 ventilator days.

**Conclusion:**

We demonstrated that by using quality improvement methodology, we successfully reduced our rate of UE by 75% to a level well below the proposed national benchmark.

## Abstract 2

Supplemental Digital Content is available in the text.

## INTRODUCTION

An endotracheal (ET) tube provides a stable airway for those critically ill neonates or children requiring mechanical ventilation and respiratory support. Unplanned extubation (UE) is the unintended removal or dislodgement of the ET tube.^1,2^ UEs are a common occurrence in critical care patients of all ages with incidences reported from 0.14 to 6.6 per 100 ventilator days.^1,3,4^ In general, neonatal intensive care units (ICU) have a higher rate than those in pediatric or adult critical care units likely due to the short length of the trachea in very small infants.^3,5,6^ Recent studies have set a target of ≤1.0 UEs per 100 ventilator days as a reasonable goal for institutions working to improve their UE rates.^5,7^ At our institution, the baseline UE rate across the children’s hospital was above this benchmark at 1.2 per 100 ventilator days.

These events can lead to significant patient harm and cost. Approximately 20% of children who experience an UE have a significant cardiovascular collapse and require cardiopulmonary resuscitation or vasoactive medications.^2,8^ In addition to potential harm, patients who experience UEs require a longer duration of mechanical ventilation and lengths of stay compared with patients with planned extubations by 2-fold in both adult and pediatric populations.^9–13^ Roddy et al.^13^ reported that patients who experienced an UE, compared with matched controls, had both increased ICU stay (10 days versus 4.5 days; *P* < 0.001) and increased total hospital stay (16.5 days versus 10 days; *P* < 0.001). Cost analysis data also demonstrate UEs correlate with approximately $36,000 increase in attributable hospital cost/per event.^13^

Reducing UEs have become a priority for many hospitals. Several studies have demonstrated that quality improvement (QI) work can reduce rates of UE within individual ICUs. Rachman et al.^14^ described a reduction in UEs of 6.4 to 1.0 per 100 ventilator days in a pediatric ICU setting, while Merkel et al.^5^ demonstrated a reduction of UEs from 2.38 to 0.58 per 100 ventilator days in a neonatal ICU. These QI efforts used education/staff awareness and process standardization to achieve their results within individual ICUs. To our knowledge, there have not been studies demonstrating uniform improvements across both pediatric and neonatal ICUs within a children’s hospital using QI methodology. We aimed to reduce the rate of UEs in our ICUs below 1 per 100 ventilator days within 2 years.

## METHODS

### Context

Yale-New Haven Children’s Hospital is a tertiary care academic children’s hospital within a larger hospital and health system. Intubated patients are managed either in the pediatric ICU or 1 of 2 neonatal ICU locations. Yale-New Haven Children’s Hospital is a level 1 trauma center with 19 pediatric ICU beds with approximately 1,100 admissions per year. It also has a level IV neonatal ICU with 54 beds at the New Haven campus with approximately 650 admissions per year and a level IIIB neonatal ICU with 20 beds at the Bridgeport campus with 200 admissions per year (referred to as a community ICU). The pediatric and level IV neonatal ICU have a fellow/advanced practice provider and in-house attendings 24/7, and they also share respiratory therapist across the main campus. The community neonatal unit is off-site from the main campus. There is an advanced practice provider overnight, and a separate group of respiratory therapists at this campus.

### Interventions

#### Formation of a Steering Committee.

A multi-professional team formed in the fall of 2014, including physicians, nurse leaders, respiratory therapists, and quality and safety leaders for the hospital. They defined the operational definition of UE as a dislodged or removed ET tube from the trachea before an order from the provider. The steering committee standardized the measurement of UEs across the units using both manual and electronic triggers and reviewed all potential events to finalize classification as either a planned or an UE. Triggers included events reported by staff during the hospital-wide morning safety report and/or in the electronic adverse event reporting system. The Steering Committee also reviewed extubations with reintubations. The electronic health record tracked total ventilation days (excluding tracheostomy days) hospital-wide and for each critical care unit. After standardization of data collection processes, the team established a baseline rate for the entire children’s hospital from January 2015 to July 2015. This shortened baseline period was due to changes in the availability of securement tape prompting the earlier need for interventions.

**Fig. 1.**
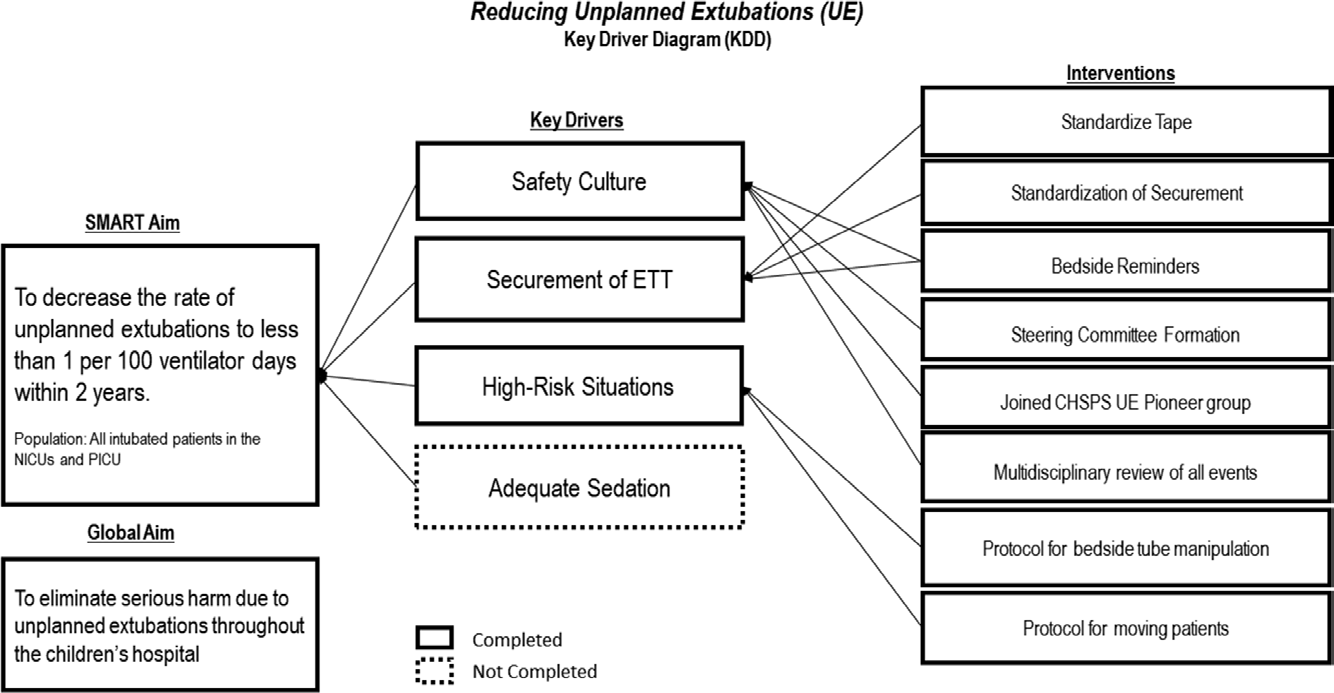
Key driver diagram outlined the aims, 4 main drivers, and interventions of the project to reduce UEs. The solid boxes are those completed, while the dotted box represents drivers not yet completed. KDD, key driver diagram; CHSPS, Children’s Hospital’s Solutions for Patient Safety.

The team defined the processes and procedures around intubation and ET tube maintenance. Prior UEs were reviewed to help categorize and prioritize common causes of UEs within our ICUs. However, events were not well documented, and many had no cause identified. Subsequently, the consensus among the multi-professional team was used to identify 4 key drivers (Fig. 1) to reduced UEs including safety culture, ET tube securement, high-risk situations, and adequate sedation. During the next 2 years, using the plan-do-study-act methodology, 8 interventions targeting the first 3 key drivers (listed below) were implemented and aimed at reducing UEs. Given the broad scope, the fourth key driver of “adequate sedation” was taken on as a separate project not directly reflected in this article.

#### Safety Culture.

In 2102, Yale New-Haven Children’s Hospital began efforts to become a high-reliability organization and promote a culture of safety. We used many of the safety culture initiatives such as event reporting, multidisciplinary review, and managing variation in this project.

### Multi-professional Review of All Events

All possible UE events that occurred during the 2-year period were peer-reviewed using apparent cause analysis. A questionnaire was completed within 24 hours of an UE by both a physician and nurse detailing any potential contributing factors, and related morbidities. Members of the steering committee reviewed and classified events and identified areas for further interventions. In addition to monthly steering committee meetings, the group also presented annually to hospital-wide leadership.

### Bedside Reminders

We tested several bedside reminders to provide quick visual cues related to the ET tube during emergent or high-risk situations. We also created a reference sheet for the standard securement methods and high-risk situation process, shown in **Supplement Digital Content 1** (available at http://links.lww.com/PQ9/A51). Other initiatives were abandoned; for instance, a doorway magnet reminder was found to scrape off paint.

### Joined Collaborative

Parallel to our hospital improvement efforts, a national collaborative was formed through the Children’s Hospitals’ Solutions for Patient Safety to reduce UEs nationally. This cohort includes over 50 children’s hospitals and works to reduce harm related to UEs across all children’s hospitals.^15^ Our hospital joined this collaborative in 2015 with support from hospital leadership. In addition to monthly steering committee meetings, leadership participated in monthly webinars with the collaborative to share ideas related to processes such as tube securement or high-risk situations. The collaborative helped reaffirm the importance of this QI work for our hospital, and provide a national network of support.

## ET TUBE SECUREMENT

### Standardization of Tape

A change in manufacturing had limited the availability of prior standard securement tape. Each critical care unit trialed several tape products during the fall of 2015. Frontline staff provided feedback related to each tape product via a short questionnaire and direct verbal feedback. After a review of the feedback, the team chose 2 products. For the pediatric ICU and community neonatal ICU, we used 3M Micropore (3M United States, St Paul, Minn.). In our level IV neonatal ICU, we used Kendall Waterproof Adhesive Tape (Cardinal Health, United States, Dublin, Ohio).

### Standardization of ET Tube Securement

After we selected tape products, standardization of a securement procedure within each unit was established and implemented. In the pediatric ICU and the community neonatal ICU, 1.0-inch tape (3M Micropore) was used to secure ET tubes less than 5 mm in size (internal diameter) with the “Y” or two “Y” taping technique. Patients requiring an ET tube equal to or greater than 5 mm in size received a commercially available device (AnchorFast Hollister Incorporated, Libertyville, Ill.) according to the manufacturer guidelines. Within our level IV neonatal ICU, a modified umbilical clip was used to anchor the tube and secured using either 0.5-inch tape for premature infants or 1.0-inch tape for full-term infants (Kendall Cardinal Health, United States). The steps involved with the tape securement methods are outlined in **Supplemental Digital Content 2** (available at http://links.lww.com/PQ9/A52). If patients transferred either from the operating room or an outside referral, staff resecured ET tubes per unit standard. Securement depth utilized anatomic references including the gum for the infant population and teeth in patients with dentition. In addition to documentation in the medical record, the securement depth, date, and nursing initials were written directly on the tape. Staff received real-time feedback during direct observation to ensure standard securement methods were used and maintained. Both nursing and physician champions helped monitor via direct observation and indirectly through verification of documentation.

## PLANS FOR HIGH-RISK SITUATIONS

### Protocol for Bedside Tube Manipulation or Moving Patients

We implemented a protocol that required 2 caregivers to participate in the identification and tracking of ET tube positioning before and after any bedside manipulation, adjustment of ET tube, or movement in any patient with an ET tube. Patient movement included bedside procedures, radiographs, patient transport, or repositioning. Repositioning defined as any movement of the midline such as the head or upper body. Extremity position changes did not require a second caregiver. A caregiver could be either a nurse, provider, or respiratory therapist. One caregiver’s sole responsibility was the protection of the ET tube. Before any movement of the patient, a caregiver performed a verbal call-out describing the depth of the ET tube. After patient movement, the location of the ET tube was again verbally confirmed. Both nurse and physician champions did a direct observation of the use of 2 caregivers.

### Study of the Intervention

The primary outcome measure was the rate of UEs per 100 ventilator days. We used statistical process control (SPC) charts and time series analysis to evaluate the interventions made. We created U-charts. Means and control limits were calculated using SPC methods that conform to U-chart primary assumptions.^16^ SPC charts developed using QI Macros software (KnowWare International, Inc. Denver, Colo.). SPC charts assessed for continual improvement, looking for both common cause and special cause variation over time.^16^ Special cause was defined as 8 consecutive points either above or below the centerline or a single point outside the control limits. We monitored compliance with interventions as a process measure using a run chart. Interventions were rolled out first in the level IV neonatal ICU as this was the largest population and was then spread to the pediatric ICU and community ICU settings. For tracking purposes, we combined the neonatal ICU populations regardless of hospital location.

### Ethical Considerations

The project was not considered as human subject research and therefore by local practices did not require review or approval from the institutional review board. We adhered to all quality-improvement ethical guidelines in the planning and implementation of this project. No interventions, compared therapies, or subjects were randomized. The study team accessed all charts and did report deidentified data to the Children’s Hospitals’ Solutions for Patient Safety collaborative. However, no personal health information was shared outside of the organization.

## RESULTS

**Fig. 2.**
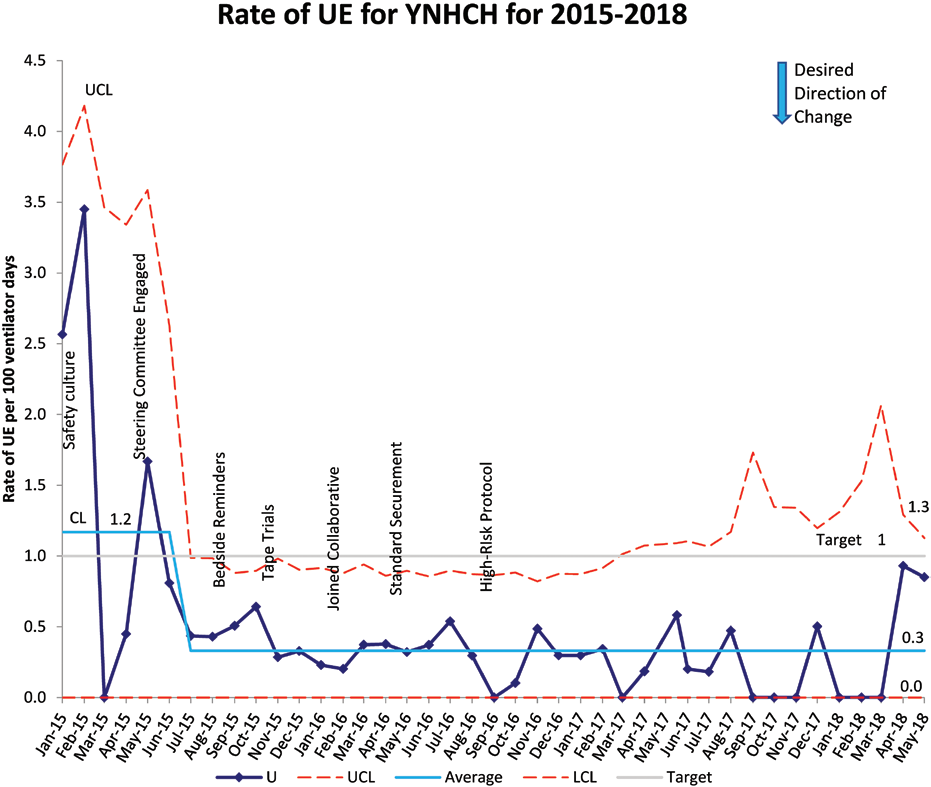
SPC chart demonstrates 8 successive points below the baseline mean during the implementation period, indicating special cause. Implementation of interventions including safety culture, bedside reminders, tape trials, joining national collaborative, standard securement, and high-risk protocols are marked. The solid line represents the baseline average, and red dotted lines represent the control limits while the gray line indicates the target rate. LCL, lower control limit; UCL, upper control limit; YNHCH, Yale-New Haven Children’s Hospital.

**Fig. 3.**
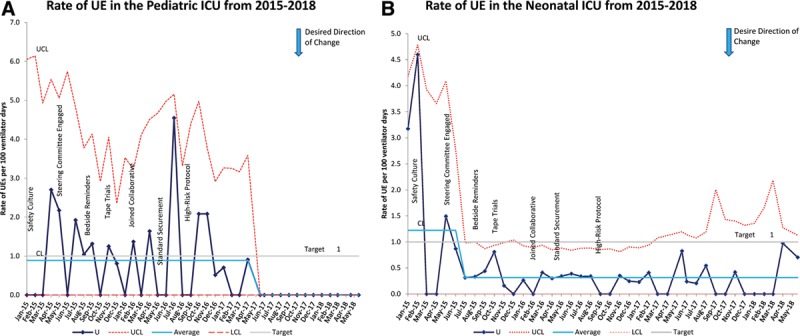
SPC charts demonstrating the rate of UEs in the pediatric (A) and neonatal (B) ICUs from 2015 to 2018. The pediatric ICU had a shift in the mean in May 2017, while the neonatal ICU had a shift early on in July 2015. The solid line represents the baseline average, and red dotted lines represent the control limits, while the gray line indicates the target rate. LCL, lower control limit; UCL, upper control limit.

From January 2015 to January 2018, there were a total of 14,558 ventilator days for the children’s hospital. We successfully reduced the rate of UEs hospital-wide by 75% from a baseline of 1.2 to 0.3 per 100 ventilator days (Fig. 2). The UE rate in the pediatric ICU decreased by 100% from 0.9 to 0 per 100 ventilator days, while the UE rate in the neonatal ICU decreased by 75% from 1.2 to 0.3 per 100 ventilator days (Fig. 3A, B). Following the baseline period, special cause variation occurred within the pediatric ICU in April 2017, when the UE rate in the pediatric ICU dropped from 0.9 per 100 ventilator days to zero. For all the ICUs, there was an improvement in practice variation as shown by the narrowed confidence limits. Shortly after interventions began in July 2015, we noted a hospital-wide shift. This shift resulted from decreased UEs within the neonatal ICUs.

The level IV neonatal ICU accounted for 70% of the mechanical ventilation days with 51% of UEs occurring within that unit. These patients all used the modified umbilical clip. The community neonatal ICU had the highest rate of UEs at 1.4 per 100 ventilatory days or 27% of UEs and only 11% of the ventilation days. Twenty-two percentage of events occurred in the pediatric ICU that made up 17% of ventilation days. Patients in the community ICU or pediatric ICU primarily used tape to secure the ET tube.

**Fig. 4.**
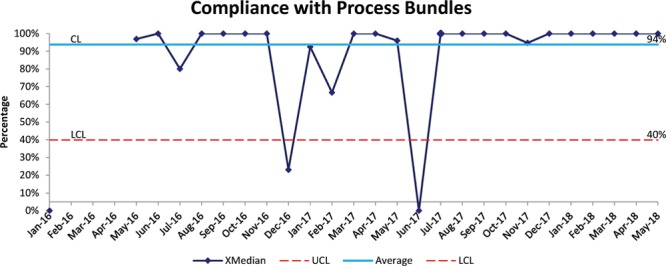
SPC chart demonstrating the overall compliance with process bundles of care related to ET tube securement and monitoring. The solid line represents the baseline average and red dotted lines represent the control limits. LCL, lower control limit; UCL, upper control limit.

Compliance with the UE process changes was collected from 2016 to 2018 and averaged 93.7% (Fig. 4). For individual bundle elements, the average compliance was 76% for standard tape securement procedures, 85.6% for high-risk situations procedures, and 98.8% for completion of UE event questionnaire. We noted special cause in December 2016, when the compliance fell below the lower control limits. This special cause reflects poor compliance with use of standard securement and use of the high-risk protocol. This degradation was after we began monitoring for high-risk situations and changed our auditing process to an electronic system. Of note, we found a dip in June 2017 when we completed no audits.

## DISCUSSION

We decreased the rate of UE in both our neonatal ICUs and pediatric ICUs using QI methodology. We sustained this improvement over 1 year. We decreased the rate by 75% to 0.3/100 ventilator days and are now well below the nationally accepted benchmark of 1 per 100 ventilator days. We estimate that we avoided 23 potential UEs, which have a high probability to cause harm. With our efforts, we have successfully brought our rate to one of the lowest rates described for both our pediatric ICU and neonatal ICU locations.^5,17–21^

The improvement found hospital-wide is largely due to the decline noted in the combined neonatal ICUs, which accounted for 82% of ventilator days. With further analysis, the community level ICU had the highest rate over the 2 years at 1.4 per 100 ventilator days. On average, the community ICU had less than 45 ventilator days per month, so one event could easily drive the rate.

One strength of the study was the implementation of improvement efforts across an entire children’s hospital system including a pediatric, community and level IV neonatal ICUs. Prior studies have focused on 1 ICU setting, and to our knowledge, this is the first to demonstrate that hospital-wide efforts could lead to sustained improvement across an entire hospital system.^1,19,22,23^ Neonatal ICUs have a higher rate of UEs compared with pediatric ICUs due to patients with smaller airways, longer intubation times, challenges using adhesive on immature skin, and lack of routine sedative and paralysis for mechanic ventilation patients.^5,19^ These challenges are similar to those with which our institution struggles. In the pediatric ICU, we routinely use continuous sedatives and soft wrist restraints to help maintain security of the ETT, while in the neonatal ICUs sedatives are more commonly given on an as-needed basis and restraints generally only used during procedures. Also, we had 3 different processes in place for tube securement. And while we did use different securement methods depending on the population of patients, the combined QI strategies allowed us to drive change across multiple ICUs.

We are one of a few institutions who has documented using a modified umbilical clip as part of the ET tube securement in the neonatal population.^24^ A prior study has shown it to be useful for securement of nasal CPAP tubing,^25^ while others such as DeJonge and White^24^ and Loughead et al.^26^ described a similar technique to ours using a modified umbilical clamp.^27^ Over 70% of ventilation days resulted from infants in the level IV neonatal ICU, where the modified umbilical clip is primarily used. It is possible that this tape securement method is one of the driving forces behind our low rate of UE within the premature population.

Overall, we suspect it was the change in culture around caring for intubated infants and children that helped drive our successful improvement. In this QI initiative, we increased vigilance and awareness of ET tube security. Starting with the formation of a steering committee (made up of leadership within each unit), we were impacting the attention and accountability of tube securement even before front-line interventions. In addition to documentation, there are visual reminders at the bedside, and tube security is frequently discussed. This practice promotes a culture of safety. Multiple studies have shown QI methodologies can successfully decrease not only UE, but improved patient transfers, reduce lengths of mechanical ventilation and overall improve patient safety,^28–30^ and that direct observation and overall promotion of a safety culture can drive change.^31,32^ This leads to systems that are highly reliably and promote quality of care, and as shown by Lyren et al.^33^ effectively reduce hospital-acquired harm.^31,34^ We feel the QI process we used reflects these core elements as highlighted by our reduced events, decreased variability within practice, and ability to track events. We sustained our improvements and recently went 12 months without an UE in the pediatric ICU, demonstrating that getting to zero harm is possible.

There are limitations to our study. First, this was a single center and the improvements accomplished locally may not be generalizable to other institutions. Second, we do not have an accurate measure of balancing measures such as resource utilization, skin integrity, or lengths of mechanical ventilation. It is conceivable that providers may have chosen to extubate patients sooner to avoid UEs, or that patients were more heavily sedated to avoid possible UEs and had a prolonged length of mechanical ventilation. We also relied on self-reporting of UE and may not have captured all events. It would be helpful moving forward to track all extubations (planned and unplanned) and lengths of mechanical ventilation as balancing measures.

Additionally, our study is limited in showing any direct benefit measures for individuals or populations. Studies show that patients with UEs have increased morbidities. We did not specifically compare outcomes such as cardiovascular collapse, duration of mechanical ventilation, or lengths of stay for patients with planned versus UEs.

## CONCLUSIONS

We demonstrated that by using QI methodology, we successfully reduced our rate of UE by 75% to a level that is well below the proposed national benchmark across a children’s hospital. By reducing these events, we prevented possible harm to our patients. These efforts followed QI methodology and are easily spread to other institutions.

## ACKNOWLEDGEMENTS

The authors acknowledge all the nursing staff and respiratory therapists across the children’s hospital for all their diligence and care toward critically ill patients. And to the patients and their families who push us to strive for zero harm continually.

## DISCLOSURE

The authors have no financial interest to declare in relation to the content of this article.

## Supplementary Material
