# Supplementary material for: Reducing Unplanned Extubations Across a Children’s Hospital Using Quality Improvement Methods
Source: Pediatr Qual Saf. 2018 Dec 11;3(6):e114. doi: 10.1097/pq9.0000000000000114 (PMC6581473; doi:10.1097/pq9.0000000000000114)

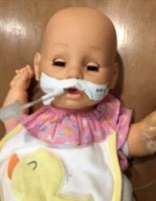


ETT less than 5.0

(PICU, Bpt Campus

NICU, CHOR, PED,

Transport)

**How to Reduce Unplanned Extubations: Fact Sheet**

***Two sets of hands are better than one!***

**1.**

**Always use 2 trained staff to tape and secure ET tubes (RN, RT, LIP, or**

**MD)**

**2.**

**Use the standard reference points when describing tube position**

•

Teeth/Gum for infants and older (PICU, PED,

Transport)

•

Lips are an acceptable reference for NNICU/NICU patients only (according to NPR guidelines)

or may be used when there are burns or trauma to the

mouth

o

**CHOR to use appropriate reference based on patients disposition*

•

Use a verbal out loud cross check to confirm placement before and after patient

transfers/moves

o

*(school age patient) “Before we move Joey, I have 13 at the teeth.”*

Patient rolled over and

repositioned.

o

*“Confirming, I see that Joey’s tube is 13 at the teeth.”*

•

Always use the standard securement process with the appropriate tape for your unit (ensure a hydrocolloid dsg

and mastisol is used as

well.)

*(Bpt Campus NICU, PICU, PED, Transport to use 3M™ Multipore; NNICU to use Covidien™ waterproof tape)*

o

**CHOR to use product and taping process based on patient’s disposition postoperatively*

•

For patients with a 5.0 tube and greater, use an Anchorfast® to secure the ETT

**3.**

**Protocol for High-Risk Situations**

•

2 trained staff must be present with one person dedicated to watch and maintain ETT security

•

Consider using the team/buddy approach if multiple patients on a particular shift require these

interventions.

***Unplanned Extubation occurred?***

Have the team complete an ACA

form after stabilizing the patient.

***Best practice:***

Complete th

e questionnaire within 60 minutes of

the event so the information is fresh in your mind!

**Thank you for all you do to keep our patients safe!**


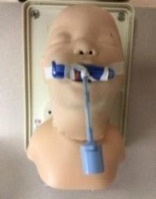


YSC

-

NNICU

**High-Risk Situations are defined as the following:**

**1.**

**Any kangaroo care, parent hold, weighing (that**

**includes moving/lifting the patient), and**

**repositioning**

•

***How do we define***

***repositioning?***

o

Anytime the midline must be moved, any head

or upper body movement (not including

extremity position

changes)

o

Examples th

at are included:

*Log rolling, changing*

*patients from prone to supine, head/neck*

*adjustments from side to*

*side*

o

Examples

NOT

included:

*Extending*

*or*

*repositioning the patient’s arm, hip or leg*

*shifting/elevating*

**2.**

**Any bedside imaging or bedside**

**procedure**

**3.**

**Any in house**

**transport**


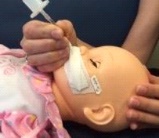


**When moving the patient ensure:**

A.

2 trained staff are

present

B.

Verbal confirmation of the tube

placement

C.

One person is “assigned” to the

ETT

D.

That the person braces the tube during

movement and ensures that there is either

enough ventilator tubing slack or disconnects

tubing to accommodate the position

change.

**Why is this important?**

Sudden, abrupt movement can dislodge the ETT.

Even gradual tugging or pressure on the tape/ETT

can cause an unplanned extubation.

When moving patients side to side, secretions can

flow out of the mouth leading to tape loosening.


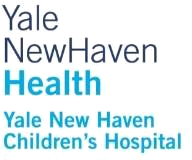

Supplement: Supplementary file 1 [file pqs-3-e114-s001.docx]
