# Supplementary material for: Reducing Unplanned Extubations Across a Children’s Hospital Using Quality Improvement Methods
Source: Pediatr Qual Saf. 2018 Dec 11;3(6):e114. doi: 10.1097/pq9.0000000000000114 (PMC6581473; doi:10.1097/pq9.0000000000000114)
Supplement: Supplementary file 2 [file pqs-3-e114-s002.docx]

1.
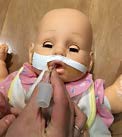
Securement using tape
   1. Place two pieces of duoderm™ on the patient’s cheeks.
   2. Apply MastisolTM on the duodermTM and on the upper lip.
   3. Allow to dry until tacky.
   4. Split two pieces of tape like trouser pants.
   5. Begin on the side of the face where the tube is and place the first piece of tape so the joint is at the corner of the mouth.
   6.
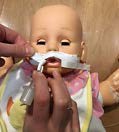
Take the top “trouser leg” and stick it across the upper lip, under the nose.
   7. Wrap the bottom “trouser leg” around the ET tube 3 times, starting close to the lips, winding up the tube in a spiral fashion maximizing adhesive contact to the ET tube.
   8. If using a cuffed ET tube, keep the cuff extension free from the tape.
   9.
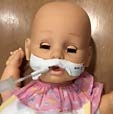
Fold over a small piece at the end in order to aid in the removal of tapes for repositioning later.
   10. Place the second piece of tape on the other side of the face so the joint is at the corner of the mouth.
   11. Wrap the top “trouser leg” around the ET tube twice, close to the lips and then secure the end of the tape on the opposite cheek.
   12. Take the bottom “trouser leg” and secure across the upper lip onto the opposite cheek.
   13. Place a dated and initialed piece of tape on the cheek/taped area.
2.
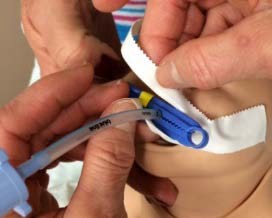
Securement using tape and modified umbilical clamp
   1. Prepare adhesive tape with “Y” cut (0.5-inch tape for preterm; 1.0-inch tape for late preterm and term)
   2. Apply Benzoin approximately the size of your tape on cheek where tape will go. Allow to dry.
   3.
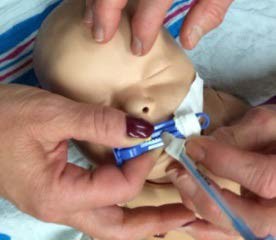
Place tape with uncut end on cheek and split end beginning just at the edge of the lip. Make sure it is sticking well to cheek.
   4. Wrap one arm of the “Y” around the clip and other arm around the ET tube
   5. Repeat taping process on opposite cheek


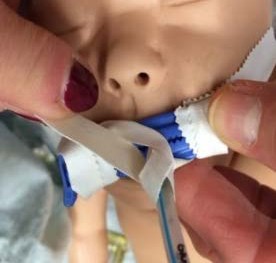
5a. Wrap opposing arm of the “Y around the clip and the other arm around the ET tube using opposite ends of respective areas

5b. Place tape with uncut end on check and split end beginning just at the edge of the lip.

- 1. Pink tape may be used to reinforce tape around ET tube and/or clip ONCE. If tape requires further reinforcement the tapes MUST BE CHANGED.
